# Case report: anti-hormonal therapy in the treatment of ductal carcinoma of the parotid gland

**DOI:** 10.1186/1471-2407-14-701

**Published:** 2014-09-23

**Authors:** Saúl Campos-Gómez, Jose H Flores-Arredondo, Rita Dorantes-Heredia, Mónica Chapa-Ibargüengoitia, Roberto de la Peña-Lopez

**Affiliations:** Departamento de Oncología Médica, Centro Oncológico Estatal, ISSEMYM, Toluca, México; Departamento de Oncología y Hematología, Instituto Nacional de Ciencias Médicas y Nutrición Salvador Zubirán (INCMNSZ), México City, México; The Methodist Hospital Research Institute, The Methodist Hospital at Houston, Houston, Texas USA; Department of Internal Medicine, Baylor College of Medicine, Houston, Texas USA; Departamento de Patología, Instituto Nacional de Ciencias Médicas y Nutrición Salvador Zubirán (INCMNSZ), México City, México; Fundación Clínica Médica Sur, México City, México; Departamento de Radiología e Imágen, Instituto Nacional de Ciencias Médicas y Nutrición Salvador Zubirán (INCMNSZ), México City, México

**Keywords:** Tamoxifen, Salivary gland, Ductal carcinoma, Estrogen receptor antagonist

## Abstract

**Background:**

Ductal carcinomas of the parotid gland are rare, highly aggressive, have a poor prognosis and are histologically similar to Ductal Breast Cancer. We report what we believe to be the first case in literature of metastatic salivary duct carcinoma (SDC) of the parotid gland with objective response to tamoxifen and aromatase inhibitors, achieving a long-term stability of disease with no associated toxicity.

**Case presentation:**

A 70-year-old female was referred to our institution for treatment of a painless nodular lesion in the scalp, localized in the frontal region of the cranium. A biopsy was taken and tested positive for metastatic ductal carcinoma. On PET CT hypermetabolic nodules were localized in the left parotid gland (11 mm), right parotid gland (10 and 12 mm), submandibular node (11 mm) and left cervical node (10 mm). A salivary ductal carcinoma was considered to be the primary tumor. The patient was subsequently started on tamoxifen, with a complete response from the scalp nodule and left parotid nodule, while the right parotid nodule demonstrated a partial response that maintained stable for 2 years until progression. Anastrazol was chosen as the next line of treatment, achieving 6 more months of stable disease. As a pseudo-adjuvant treatment, surgical resection of the right parotid lesion was performed and helped achieve two years of disease stability.

**Conclusions:**

Estrogen receptor antagonists such as tamoxifen or aromatase inhibitors may represent a target for the establishment of a safe alternative and novel therapy for SDC, however more accurate data obtained from larger studies are required.

## Background

Ductal carcinomas of the parotid gland are rare, highly aggressive, have a poor prognosis and are histologically similar to Ductal Breast Cancer. Salivary gland tumors metastasize most frequently to lymph nodes, lung, liver and bones. Distant metastases occur in 27%, independent of histology and 46% in adenoid cyst cell types and high-grade salivary ductal carcinoma [1]. Surgical resection of solitary metastasis may be considered in select cases, but the goal of chemotherapy in metastatic or recurrent disease is palliative care, based on response rates that range from 10-30% and the lack of evidence benefiting survival [2].

Salivary ductal carcinomas are an uncommon and high-grade adenocarcinoma arising from the ductal epithelium, which Kleinsasser et al. reported in 1968 for the first time [3], and the World Health Organization classified as a distinct neoplasm in 1991 [4]. Ductal carcinomas, typically present in the sixth or seventh decade of life and are more prevalent in males (3:1.8) [5, 6]. The parotid gland is involved in 80% of diagnosis, followed by the submandibular gland in 8% and the rest in the minor salivary glands [7, 8]. The aggressive behavior characterizing ductal carcinomas is associated with rapid progression, early lymph node metastasis, a high risk of local recurrence, distant metastasis and ultimately a low survival rate, 3 yrs. median [9, 10].

Due to SDC’s histological similarity to ductal carcinoma of the breast, hormonal receptor status and regulation has been a subject of interest. However, hormone receptor expression is virtually absent in most malignant salivary gland tumors, with the exception of the androgen receptor, which is present in approximately 92% of cases. Similar to invasive ductal carcinoma of the breast, overexpression and amplification of the HER-2 gene is present in SDC. Patients with SDC and HER-2 overexpression and amplification may be targeted with trastuzumab and obtain positive responses to therapy [8]. SDC in comparison to ductal carcinoma of the breast expresses in a lower percentage estrogen (−8%) or progesterone receptors [9]. Androgen receptor (AR) therapy however has emerged as a possible target, due to the fact that a majority of SDC lesions express AR and have demonstrated clinical benefit [10].

Cytotoxic chemotherapy has limited benefit in a subtype of SDC that is rare and aggressive, for which genotyping analysis has been pursued to help identify novel tumor-specific mutations that may help direct targeted therapies in these cases. There has been an interest in recently discovered PIK3CA, PTEN and BRAF V600E kinase mutations in subsets of HER2-negative SDC; due to these recent discoveries the aforementioned pathways have been suggested as therapeutic targets [11, 12].

The following case report is what we believe to be the first case in literature of metastatic salivary duct carcinoma of the parotid gland with objective response to tamoxifen and aromatase inhibitors, achieving a long-term stability of disease with no associated toxicity.

## Case presentation

**Figure 1:**
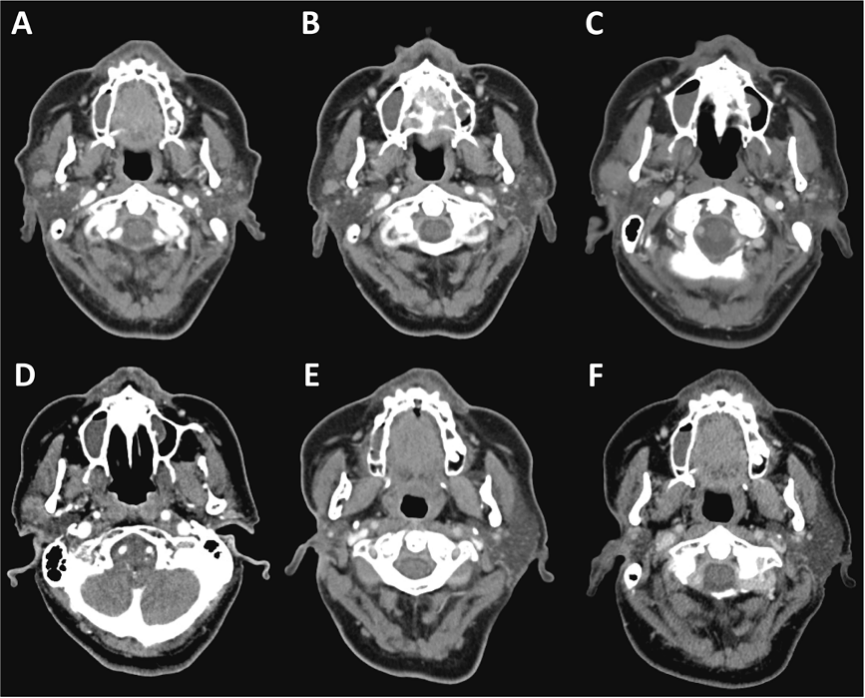
**Computed Tomography (CT). A** CT showing solid lesion in right parotid gland (Baseline), **B** Stable Disease under tamoxifen treatment. **C** Growth of the solid lesion in right parotid, **D** Stable Disease after switch to anastrazol, **E** Postsurgical CT showing absence of right parotid gland **F** Stable disease in surveillance.

**Figure 2:**
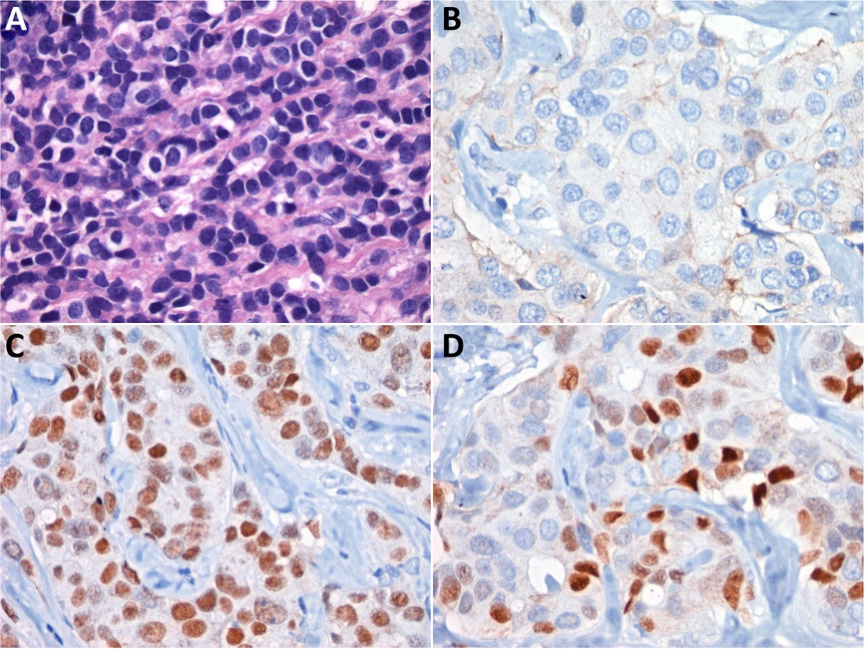
**Haematoxylin**-**eosin stain (H & E) and Immunohistochemical studies. A** Salivary Duct Carcinoma of Parotid Gland with cribriform growth pattern (H & E), **B**. Her2/neu immunostaining showing negative membrane staining, **C** Positive Estrogen receptor immunostain, **D** Positive Progesterone receptor immunostain.

A 70-year-old female was referred for treatment of a painless nodular lesion with a diameter of 2 cm in the scalp, localized in the frontal region of the cranium. Her past medical history was unremarkable. A biopsy was taken and tested positive for metastatic ductal carcinoma. Further analysis revealed Estrogen Receptor positivity. Mastrography and CT did not demonstrate any evidence of a primary tumor, while PET CT showed hypermetabolic nodules localized as follows: 11 mm in the left parotid gland, 10 and 12 mm in the right parotid gland, 11 mm submandibular node and 10 mm left cervical node. At this point, there was clinical suspicion of a primary salivary ductal carcinoma. The patient was started on tamoxifen, 20 mg daily, with a complete response in the scalp nodule and left parotid nodule, although a partial response was observed on the right parotid lesions (Figure 1B). The patient continued stable for two years, after which progression of the right parotid nodule was observed through clinical palpation and through CT as a lesion 2 cms in diameter located in the right periaricular area (Figure 1C), without cranial nerve involvement or palpable cervical lymphadenopathy. There was no evidence of any metastatic lesions. Anastrozole 1 mg/day was started, achieving stable disease within 8 months of treatment (Figure 1D). Local control of the parotid lesion was obtained via surgical resection (Figure 1E), tissue was sent to pathology, where a Hematoxylin and Eosin (H & E) stained section showed ductal structures (Figure 2A). Immunohistochemistry was performed using standard procedures with monoclonal antibodies: 6 F11 (Novacastra Laboratories Ltd., Burlingame, CA) for ER and 1A6 (Novacastra Laboratories Ltd., Burlingame) for PR and AB-8 monoclonal antibody (NeoMarker Lab Vision Co., Fremont, CA) for Her2, which proved negative for Her2neu and positive for Estrogen and Progesterone Receptors (Figure 2B, C, D). The tumor was classified as a salivary duct carcinoma of the parotid gland. At this time, the patient had been receiving anastrozole as pseudo adjuvancy for two years without further evidence of disease (Figure 1F).

## Conclusions

The histologic similarities between salivary duct carcinoma and ductal carcinoma of the breast in addition to the low response rates to chemotherapy have led to antihormonal therapies being considered for treatment. Salivary duct carcinomas responding to antiandrogen therapies and advanced adenoid cystic salivary tumors to Tamoxifen have been reported in the literature [13, 14]. However, Tamoxifen and Aromatase inhibitors (estrogen signaling inhibitor agents) have not been reported in the literature, due to the low expression of ER in Ductal Carcinoma of the Salivary Gland.

Tamoxifen and anastrazole have proven to be effective in Ductal Carcinoma of the Breast and were considered a potential novel therapy in this case, stopping the progression of salivary gland carcinoma (SGC) and achieving long-term stability of ductal parotid carcinoma. Tamoxifen prevents the activation of estrogen responsive genes by inhibiting both translocation and nuclear binding of the receptor itself. Aromatase inhibitors like Anastrozole on the other hand prevent ER activation by inhibiting the conversion of androgens to estrogens by binding to the aromatase enzyme.

Due to limited management options in salivary gland carcinoma and low rates of response to chemotherapy the goal of treatment for metastatic salivary gland carcinoma is palliation, since there is not strong evidence that survival is prolonged with systemic treatment. Targeted cancer therapies represent a promising strategy to treat these rare aggressive malignancies. Albeit, the expression of sex steroid hormone receptors in salivary gland carcinoma and the evidence of linking hormone receptors and growth factor receptors to the disease may lead to the use of hormone therapy in these specific cases. Estrogen receptor antagonists such as tamoxifen or aromatase inhibitors may represent a target for the establishment of a safe alternative therapy for SDC but more accurate data obtained from larger studies are required.

Genetic profiling is a valuable approach to identify actionable mutations (BRAF or PIK3CA mutations) which could have a significant impact in this rare in highly aggressive malignancy.

### Consent

Written informed consent was obtained from the patient for publication of this case report and any accompanying images. A copy of the written consent is available for review by the Editor of this journal.

## Abbreviations

SDC: Salivary Ductal Carcinoma
EGFR: Epidermal growth factor receptor
ER: Estrogen Receptors
H & E: Hematoxylin and Eosin
SGC: Salivary gland carcinoma.
